# Supplementary material for: Evaluation of Helium Ion Radiotherapy in Combination with Gemcitabine in Pancreatic Cancer In Vitro
Source: Cancers (Basel). 2024 Apr 14;16(8):1497. doi: 10.3390/cancers16081497 (PMC11049166; doi:10.3390/cancers16081497)
Supplement: Supplementary file 1 [file cancers-16-01497-s001.zip › cancers-2937426-supplementary.pdf]

Table S1: Evaluation of the therapeutic effect for each dose after combination of gemcitabine treatment with photon irradiation. We abbreviated every single dose as RT(dose1)ChT(dose2) : Dose 1 shows the radiation dose and dose 2 the gemcitabine dose.

**AsPC-1**	RT2ChT10	independent
	RT4ChT10	independent
	RT6ChT10	independent
	RT2ChT50	additive
	RT4ChT50	additive
	RT6ChT50	independent
**BxPC-3**	RT2ChT10	independent
	RT4ChT10	additive
	RT6ChT10	independent
	RT2ChT50	additive
	RT4ChT50	additive
	RT6ChT50	additive
**Panc-1**	RT2ChT10	independent
	RT4ChT10	independent
	RT6ChT10	independent
	RT2ChT50	independent
	RT4ChT50	independent
	RT6ChT50	independent

Table S2: Evaluation of the therapeutic effect for each dose after combination of gemcitabine treatment with helium ion irradiation. We abbreviated every single dose as He(dose1)ChT(dose2) : Dose 1 shows the helium ion radiation dose and dose 2 the gemcitabine dose.

**AsPC-1**	He1ChT10	additive
	He2ChT10	additive
	He3ChT10	independent
	He1ChT50	additive
	He2ChT50	additive
	He3ChT50	independent
**BxPC-3**	He1ChT10	additive
	He2ChT10	additive
	He3ChT10	additive
	He1ChT50	additive
	He2ChT50	additive
	He3ChT50	additive
**Panc-1**	He1ChT10	independent
	He2ChT10	independent
	He3ChT10	independent
	He1ChT50	additive
	He2ChT50	additive
	He3ChT50	additive
